# The Multifaceted Aspects of Clinical Nutrition—An Introduction to Hot Topics in Clinical Nutrition (2nd Edition)

**DOI:** 10.3390/nu16183182

**Published:** 2024-09-20

**Authors:** Emanuele Rinninella, Costanza Ceci, Antonio Gasbarrini

**Affiliations:** 1 Department of Translational Medicine and Surgery, Università Cattolica Del Sacro Cuore, Largo F. Vito 1, 00168 Rome, Italy; antonio.gasbarrini@unicatt.it; 2 Research and Training Center in Human Nutrition, Catholic University of the Sacred Heart, 00168 Rome, Italy; 3 Clinical Nutrition Unit, Department of Medical and Abdominal Surgery and Endocrine-Metabolic Sciences, Fondazione Policlinico Universitario Agostino Gemelli IRCCS, Largo A. Gemelli 8, 00168 Rome, Italy; 4 Degree Course in Pharmacy, Catholic University of the Sacred Heart, 00168 Rome, Italy; costanza.ceci01@icatt.it; 5 Digestive Disease Center (CEMAD), Department of Medical and Abdominal Surgery and Endocrine-Metabolic Sciences, Fondazione Policlinico Universitario Agostino Gemelli IRCCS, 00168 Rome, Italy

Clinical nutrition plays a pivotal role in several areas of medicine and has a significant impact on patient outcomes in both acute and chronic conditions. Recent advances in nutrition research have revealed new links between diet and health and have proposed increasingly targeted tools and personalised dietary interventions to improve diagnosis, treatment and patient management at every stage of human life. In this special issue, we have brought together excellent contributions from different areas of clinical nutrition. This unique mix of ideas and evidence provides the reader with the opportunity to briefly review the latest evidence on most of the hot topics in clinical nutrition. The issue consists of nine original articles and two narrative reviews.

Much attention is being paid to sarcopenia, a progressive and generalized skeletal muscle disorder characterised by low muscle strength, low muscle quantity or quality, often leading to low physical performance [1]. Primary sarcopenia is often associated with ageing; secondary sarcopenia may be the result of a catabolic state due to acute or chronic processes, such as malignancy or organ failure. In both cases, it may be an independent risk factor for mortality and morbidity [2,3]. Successful management of sarcopenia is difficult in clinical practice due to the many challenges that need to be addressed (nutritional needs, physical inactivity and inflammatory processes). As far as we know, low levels of plasma proteins, vitamin D and omega-3 polyunsaturated fatty acids (*n*-3 PUFA) are associated with sarcopenia. On the other hand, carnitine may play a key role in skeletal muscle cell metabolism, being involved in the transport of fatty acids across the inner mitochondrial membrane for ß-oxidation. However, the role of these factors in sarcopenia remains to be elucidated and serological biomarkers for early diagnosis of sarcopenia are lacking. Ho et al. [4] proposed a metabolomic approach to study the plasmatic profiles of elderly (>65 years) Taiwanese patients followed for a median of 5.5 years. They found that low levels of DHA-containing phosphatidylcholine diacyl (PCaa) C38:6 and high levels of tetradecanoyl carnitine (C-14 carnitine) correlated with increased mortality risk, suggesting a novel plasma metabolic signature of higher mortality in sarcopenic patients. Research on *n*-3 PUFA has highlighted their role in normalizing mitochondrial dysfunction, as demonstrated in various cell types and tissues in experimental models of organ dysfunction [5,6]. In this issue, Gortan Cappellari et al. [7] demonstrated that dietary supplementation with *n*-3 PUFA can normalize mitochondrial dysfunction induced by chronic heart failure (CHF) while preserving muscle mass in a rodent model. This study provides novel translational insights into the understanding of muscle inflammatory disorders with potential implications for clinical practice. One of the main causes of sarcopenia and malnutrition in cancer patients is low energy intake due to cancer anorexia. Anorexia can occur in up to 70% of cancer patients [8,9]. Cancer itself and the immune response can induce metabolic derangements leading to anorexia; a new field of investigation is the autonomic nervous system, given its potential influence on anorexia and its contribution to weight loss. Molfino et al. [10] investigated the dysregulation of the autonomic nervous system in cancer patients with and without anorexia (compared to healthy controls) by examining heart rate variability (HRV). The results showed a reduction in both sympathetic and parasympathetic activity, which correlated with weight loss in cancer patients with anorexia.

Another challenge in clinical nutrition is the management of chronic intestinal failure (CIF), which is defined as “the prolonged reduction of intestinal function below the minimum required for nutrient and/or water/electrolyte absorption, requiring intravenous supplementation to maintain body health and function and nutritional homeostasis” [11]. CIF is often the clinical consequence of short bowel syndrome (SBS) resulting from colon resections for various conditions such as ischaemic, neoplastic, inflammatory, or congenital bowel disease. Patients with SBS experience reduced absorption and significant fluid loss from their stoma. Two gut hormones, glucagon-like peptide (GLP)-1 and GLP-2, which play an important role in gut homeostasis, are deficient in SBS. The first (GLP-1), delays gastric emptying, prolongs intestinal transit, and promotes satiety at the central nervous system (CNS) level. The second (GLP-2), promotes villous growth and improves transepithelial absorption. While GLP-2 analogues (e.g., teduglutide) have been tested as promising agents in SBS [12], the role of GLP-1 agonists, which are widely used in the treatment of diabetes mellitus, in SBS is still largely unknown. Merlo et al. [13] conducted a real-life observational study in an Italian tertiary referral center for CIF support in SBS patients, describing the effects of liraglutide (a GLP-1 agonist) at 1 and 6 months in 19 adults (non-cancer) patients with a new diagnosis of SBS within 1 month of surgical resection. The Authors showed a significantly reduced stool and stoma output in treated patients compared to untreated patients. This study highlights how new pharmacological treatments could enhance patients’ quality of life and nutritional status in SBS, reducing the need for repeated PN and optimizing overall SBS management.

However, total parenteral nutrition (TPN) is one of the mainstays of clinical nutrition in certain settings. Optimization of AN, both enteral and PN, is crucial at every stage of human life, especially in the premature neonate, where the need for catch-up growth must be balanced against intestinal immaturity and the risk of intravenous bloodstream infections. The review by De Rose et al. [14] covers a wide scenario of potentially harmful and life-threatening situations that arise in the care of preterm infants in the Neonatal Intensive Care Unit (NICU), where nutritional practices do not always follow evidence-based standards based on guidelines. This review provides useful insights by reporting on best evidence-based practice in several topics such as the use of standard versus individualized parenteral nutrition, the evolution of enteral nutrition (EN) in preterm infants, the role of human milk and milk fortification in preterm infants undergoing surgery, with a special focus on neonates at risk of necrotizing enterocolitis (NEC).

Adherence to a strict dietary regimen is essential for achieving and maintaining normal metabolic health even at other stages of childhood development and in adulthood, particularly in people affected by inborn errors of metabolism (IEMs). IEMs are a heterogeneous group of rare genetic disorders in which a defect in a biochemical pathway—a primary enzyme defect or cofactor deficiency—results in the deficiency or accumulation of one or more metabolites, leading to organ dysfunction [15]. The most common disorders result from genetic defects in enzymes or cofactors involved in the metabolism of amino acids-e.g., phenylketonuria (PKU), maple syrup urine disease (MSUD), homocystinuria, tyrosinemias, urea cycle disorders (UCDs)-, carbohydrates (e.g., galactosemia, hereditary fructose intolerance, glycogen storage disease) and fatty acids (e.g., fatty acid b-oxidation defects). Personalized dietary interventions are often lifesaving, helping to prevent the accumulation of toxic intermediates and long-term complications. Educational tools for patients and their families are needed to maintain dietary compliance and promote autonomy and food enjoyment. In 2008, a free online dietary calculator called Odimet^®^ was developed, providing details of over 3000 foods, including those specific to IEM. A study by Sánchez-Pintos [16] and colleagues analyzed the use of Odimet in 120 patients over three periods before and after the COVID-19 pandemic. Results showed good metabolic control in pediatric and adult patients, with adequate levels of phenylalanine, leucine, glutamine, and galactose-1-phosphate. The program includes 14,825 products and will support 63 emergency dietary adjustments in 2023. However, while an education program can effectively maintain adherence, the poor taste of current dietary and pharmaceutical products can make lifelong enzyme therapy or cofactor replacement a challenge for some patients, particularly children. In these cases, the use of 3D printing technology and artificial intelligence (AI), which is currently being evaluated, has enabled the creation of personalized dosage forms that overcome the main limitations of current therapies and offer numerous advantages for improving adherence, safety, and treatment efficacy. The review by Carou-Senra et al. [17] addresses key aspects of this important topic, with particular emphasis on Semi-Solid Extrusion (SSE) technology. This specialized technique allows the deposition of gels, waxes, or pastes to produce solid objects at low temperatures, facilitating the development of chewable formulations with different shapes, colors, flavors, and textures. These formulations have shown high acceptance, particularly in pediatric patients.

Despite being well metabolized, many other foods or dietary antigens can cause adverse food reactions (AFRs) in many people, whose symptoms are often associated with irritable bowel syndrome (IBS), celiac disease (CD), non-celiac gluten sensitivity (NCGS) and nickel allergic contact mucositis (Ni-ACM). Many patients also complain of systemic manifestations such as neurological, dermatological, joint, and respiratory disorders. The diagnostic and therapeutic approach can be challenging, resulting in wasted time and frustration for patients and clinicians. Advanced diagnostic tools such as the oral mucosal patch test for gluten (GOMPT) and nickel (Ni-OMPT), supported by immunohistochemical analysis, may improve the correct identification of such diseases. An observational study by Greco et al. [18] addresses this issue and proposes a useful diagnostic algorithm.

On the other side, other nutrients and foods can be considered healthy for everyone: micronutrients and botanical supplements have an important role to play in both prevention and treatment, supporting overall health through their antioxidant and anti-inflammatory properties [19]. This may also apply to inflammatory and degenerative conditions such as vascular disease and cognitive impairment. Following this rationale, Wang et al. [20] conducted a randomized controlled trial in which healthy participants received a dietary intervention of fresh blueberries, freeze-dried blueberry powder, or placebo for one week, searching for individual differences in response using endpoints related to vascular health and cognitive decline.

Dietary personalization should also take into account the modulation of the gut microbiota, which is a very hot topic because of its role in immunomodulation and non-communicable diseases. There is a large body of evidence on dietary modulation of the gut microbiota [21]. Less is known about the effect of surgery on the gut microbiota. Current research suggests that cholecystectomy (CCE) may affect gut microbiota homeostasis due to altered bile acid (BA) metabolism. This has been implicated in the development of non-alcoholic fatty liver disease (NAFLD), colorectal cancer (CRC), and cardiovascular disease. Xu et al. [22] meta-analyzed 218 raw 16S rRNA gene sequencing datasets to determine consistent patterns of structural and functional changes in the gut microbiota after CCE, finding significant differences between the microbiota of CCE and the control group. Moreover, fecal calprotectin levels were significantly higher, and short-chain fatty acids (SCFAs) decreased in the CCE group, confirming that CCE exposes these patients to a higher risk of intestinal inflammation. The authors also confirmed how a high-fat diet (following CEE) could enhance the inflammatory milieu of the gut ecosystem. The study highlights the need for targeted nutritional strategies to maintain gut balance and improve outcomes post-surgery.

In recent decades, physical activity has gained attention for its role in reducing the burden of inflammatory diseases. A study by Gavilán-Carrera [23] al investigated the effects of a 12-week aerobic exercise intervention on body composition and adherence to the Mediterranean diet in women with systemic lupus erythematosus (SLE).

In conclusion, the ongoing evolution of nutritional strategies offers a promising outlook for a future in which disease management will become increasingly precise and effective. The integration of advanced technologies and personalized nutritional approaches represents a significant advance that not only improves patients’ quality of life but also provides tools for optimal disease management, reducing complications and promoting longer-term patient well-being.
